# Importance of Polarity Reversal in Leads I/aVL in the Diagnosis of an Accessory Pathway Originating from the Aortomitral Continuity

**DOI:** 10.19102/icrm.2021.120604

**Published:** 2021-06-15

**Authors:** Enes Elvin Gul, Sohaib Haseeb

**Affiliations:** ^1^Division of Cardiac Electrophysiology, Madinah Cardiac Centre, Madinah, Saudi Arabia; ^2^College of Medicine and Dentistry, James Cook University, Townsville, Queensland, Australia

**Keywords:** Aortomitral continuity, accessory pathway, catheter ablation, electrocardiogram

## Abstract

Accessory pathways (APs) are commonly located around the tricuspid and mitral annulus; however, they can be rarely seen in unusual locations like the aortomitral continuity (AMC), the right atrium to the right ventricular outflow region, and the left atrial appendage to left ventricle connection. Although several electrocardiogram algorithms have been proposed to localize the AP, the sensitivity of these algorithms is not high and they may fail to localize the mentioned unusual localizations. In this report, we describe a case of a 37-year-old man presenting with an AP originating from the AMC, which was successfully ablated.

## Case presentation

**Figure 1:**
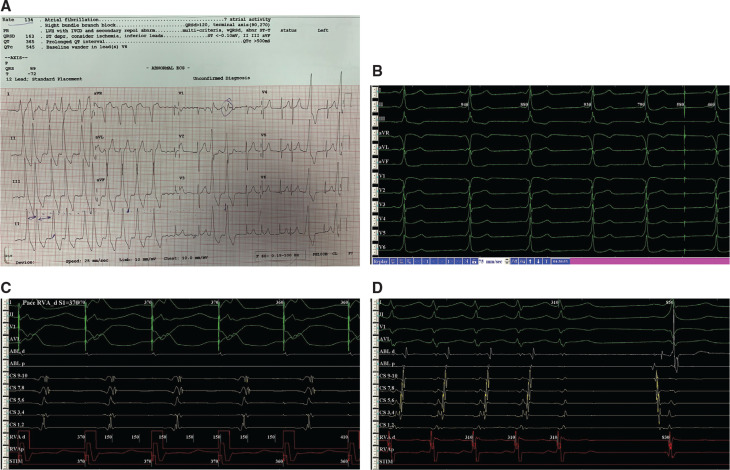
**A:** Initial ECG showing a Wolff–Parkinson–White pattern and AF. Note a positive delta/QRS wave in lead I and negative delta/QRS wave in lead aVL as well as qR in lead V1 and Rs in lead V2. **B:** ECG in sinus rhythm showing the same reverse polarity in leads I and aVL. **C:** Retrograde atrial activation sequence during right ventricular apical pacing showing eccentric activation with earliest atrial signals in CS 1–2. **D:** Nonsustained AV reentrant tachycardia with eccentric activation (earliest A in distal CS). Also, note late ventricular activation in the His channel relative to the far-field ventricular signal in distal CS (sinus beat following the tachycardia termination).

A 37-year-old man with a medical history of previous failed catheter ablation 10 years earlier presented to the emergency department with syncope. A surface 12-lead electrocardiogram (ECG) done in the emergency department showed overt preexcitation and atrial fibrillation (AF) **(Figure 1A)**. Due to hemodynamic compromise, the patient required urgent electrical cardioversion. He was subsequently admitted to the cardiology unit for consideration of an electrophysiology study and radiofrequency catheter ablation.

Twelve-lead ECG analysis of preexcited sinus rhythm showed positive delta waves in leads I, II, III, and aVF. The delta wave in lead aVL was biphasic and negative in aVR. Precordial leads showed a negative delta wave in V1 and positive delta wave in V2 **(Figures 1A and 1B)**. These ECG findings were compatible with rightward anterior and superior localization of the accessory pathway (AP). The mentioned ECG findings suggested the existence of a para-Hisian or septal AP.

The patient was taken to the electrophysiology laboratory and a three-dimensional mapping system (CARTO 3®; Biosense Webster, Diamond Bar, CA, USA) was used due to suspicious localization of the AP carrying a high risk of atrioventricular (AV) nodal injury. In addition, the cryoablation system was also made ready for the procedure. Initially, catheters were placed on the coronary sinus (CS), right ventricle, and His region. An electrophysiology study was performed and the effective refractory period of the AP (APERP) was recorded at 270 ms. AF with preexcitation was easily induced and the shortest preexcited R–R interval was measured as 240 ms. Due to a history of syncope as well as the high-risk properties of the AP, catheter ablation was considered very crucial. The right atrium (RA) was first mapped and no earliest points were found. Activation mapping was performed during atrial pacing at 500 ms from the proximal CS. A ventricular signal in the His region was noted as later than the far-field ventricular signal in CS 1–2 during atrial pacing from the proximal CS. Retrograde pacing demonstrated eccentric activation, with the earliest atrial signal existing at the distal CS 1–2 **(Figure 1C)**. Activation mapping of the RA failed to show any of the earliest ventricular insertion points. Nonsustained tachycardia (AV reentrant tachycardia) with eccentric activation was also inducible **(Figure 1D)**. No attempts of catheter ablation were made in the right side, close to the anteroseptal region.

**Figure 2:**
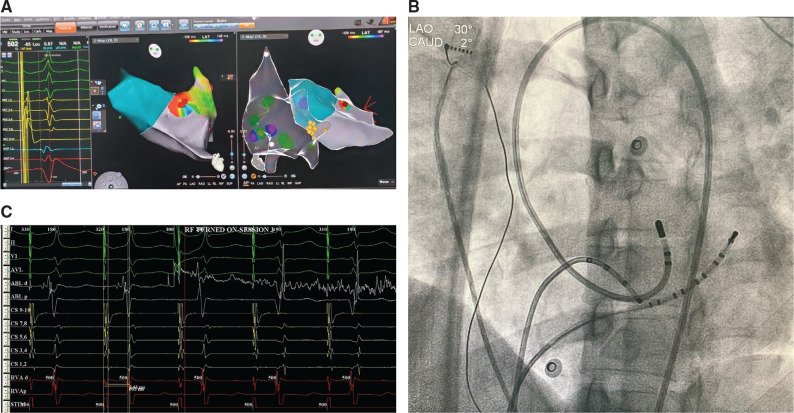
**A:** Activation mapping performed during atrial pacing from the proximal CS showing earliest signals in the AMC region and successful radiofrequency energy applications (red dots). His signals are also seen (yellow points). Note the earliest V signal in the map 1–2 catheter. **B:** Fluoroscopy in left anterior oblique projection showing the ablation catheter positioned in the AMC via the retrograde transaortic approach at the site of successful ablation of the AP. **C:** Radiofrequency ablation successfully eliminated the AP within 1.4 seconds.

**Figure 3:**
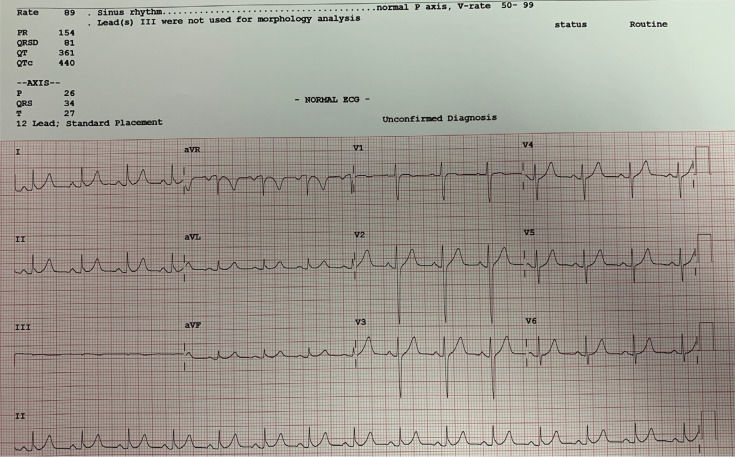
Final 12-lead ECG showing sinus rhythm without any evidence of the AP. Note the early repolarization pattern in the inferior leads.

Due to the positive delta wave in lead I, we decided to map the coronary cusps (particularly the right coronary cusp and noncoronary cusp via a retroaortic approach first. No remarkably early points were found around the coronary cusps. Then, mapping of the lateral mitral annulus was performed. Slightly earlier points were found here; however, the signals were not very impressive. Septal aspects of the tricuspid annulus, the mitral annulus, and aortic coronary cusps did not show any earlier points. Therefore, the only nonmapped region was the aortomitral continuity (AMC). Positioning the mapping/ablation catheter just below the aortic valve revealed very early ventricular signals, and radiofrequency application at this location eliminated AP immediately within 1.2 seconds **(Figure 2)**. After a waiting period of 30 minutes, 12 mg of adenosine was given intravenously and no evidence of antegrade or retrograde conduction over the AP was observed. The final 12-lead ECG showed sinus rhythm with no signs of preexcitation **(Figure 3)**. The patient was discharged the same day with directions to take 100 mg of acetylsalicylic acid for four weeks.

## Discussion

AV APs are considered absent from the fibrous AMC, although recent reports have described very few patients who had undergone AP ablation at this location.^1–3^ A clue to this type of AP is that if the preexcitation pattern suggests an anteroseptal location but the local ventricular activation in the anteroseptal area is either not early or is far-field, mapping at or below the coronary cusps is recommended.

Traditional ECG algorithms to localize APs are used in daily practice.^4,5^ Although the sensitivity and specificity of these algorithm do not reach 100%, a preliminary diagnosis in our case in favor of an anteroseptal AP was made. However, the electrophysiology study demonstrated earliest activation in the left side. The only clue against anteroseptal AP was the presence of a negative delta/QRS axis in lead aVL. Interestingly, previous reports in the literature **(Table 1)** included mentions of negative delta/QRS waves in aVL, a feature that distinguishes them from septal APs.^1–3^ Algorithms were not helpful for localization in our case nor in these previous reports. Early transition in V2, a negative delta/QRS axis in the lead aVL, and a positive delta/QRS axis in lead I suggested the localization of AP between the left lateral/anterolateral mitral annulus and the anteroseptal region.^4,5^ In reality, the AP existed between these two locations, where it fits with the concept of AMC, which is a very unusual location for APs.

Interestingly, no previous authors to our knowledge have mentioned polarity reversal in leads I and aVL. In our case and in previous cases, QRS polarity was positive in lead I and negative in lead aVL, which is not compatible either with an anteroseptal AP or left lateral mitral annulus AP.^1–3^ Therefore, we suggest that polarity reversal (positive QRS in lead I and negative QRS in lead aVL) might strongly indicate the location of the AP in the AMC. However, studies with more patients are required to clarify the sensitivity and specificity of this ECG finding.

Another interesting ECG finding in our case was the presence of a QS pattern in lead I during maximal preexcitation in AF. Theoretically, a similar QRS morphology should be observed in patients with premature ventricular complexes from the AMC, composed of the following aspects: qR in V1, deep S-wave in V2, negative QRS in leads aVR and aVL, and an inferior axis (positive QRS in inferior leads).^6^

## Conclusion

Here, we discuss for the first time the importance of considering a reverse QRS polarity in leads I and aVL in predicting the location of APs originating in the AMC, which is a site where APs are not usually thought to be present.

## Figures and Tables

**Table 1:** Clinical, Electrocardiographic, and Electrophysiology Characteristics of AP Cases Using AMC in the Literature

	Present case	Jastrzebski et al.^1^	Liew et al.^2^		Kupó et al.^3^
			Case #1	Case #2	
Age (years)	37	36	24	27	40
Sex	M	M	F	F	M
Past medical history	Failed catheter ablation	Abandoned catheter ablation due to high-risk of AVN injury	None	None	NA
Presentation	Syncope and preexcited AF	Palpitation	Palpitation	Palpitation	Palpitation
APERP and SPRRI (ms)	270 and 240	280 and N/A	N/A	N/A	N/A
Delta/QRS direction					
Lead I	Positive	Positive	Positive	Positive	Positive
aVL	Negative	Negative	Negative	Negative	Negative
II/III/aVF	Positive	Positive	Positive	Positive	Positive
V1	Negative	Positive	Positive	Negative	Negative
V2	Positive	Positive	Positive	Negative	Positive
V1/V2	< 1	< 1	< 1	< 1	< 1
Catheter ablation					
Successful	Yes	Yes	Yes	Yes	Yes
Approach	Retroaortic	Retroaortic	Transeptal	Retroaortic	Transeptal

AP: accessory pathway; AF: atrial fibrillation; AMC: aortomitral continuity; AVN: atrioventricular node; APERP: accessory pathway effective refractory period; M: male; N/A: not applicable; SPRRI: shortest preexcited R–R interval.
